# Sex Differences in Depression: Adult Cytogenesis as Potential Target for Precision Psychiatry

**DOI:** 10.3390/cells15121059

**Published:** 2026-06-10

**Authors:** Leandro Rodrigues-Freitas, Luísa Pinto, Teresa Canedo

**Affiliations:** 1 Life and Health Sciences Research Institute (ICVS), School of Medicine, University of Minho, 4710-057 Braga, Portugal; 2 ICVS/3B’s—PT Government Associate Laboratory, 4710-057 Braga, Portugal; 4806-909 Guimarães, Portugal; 3 Bn’ML—Behavioral and Molecular Lab, 4710-057 Braga, Portugal

**Keywords:** sex differences, depression, adult neurogenesis, adult astrogliogenesis

## Abstract

**Highlights:**

**What are the main findings?**

- Sex differences in depression arise from the interaction of multiple biological factors, including chromosomal sex, gonadal hormones, HPA axis reactivity, brain structure, and cellular plasticity, with the hippocampus emerging as a key integrative hub.
- Adult cytogenesis, encompassing both neurogenesis and astrogliogenesis, is altered in stress-induced depression in a sex-dependent manner, with males and females displaying distinct cellular and behavioral responses to stress.

**What is the implication of the main finding?**

- Recognizing biological sex as a fundamental variable in neuroscience and psychiatry research is crucial for uncovering mechanisms that contribute to depression and for improving diagnosis and treatment strategies.
- By shaping hippocampal plasticity, adult cytogenesis represents a promising avenue for precision psychiatry, opening the possibility for sex-tailored interventions aimed at restoring brain function in depression.

**Abstract:**

Sex differences are increasingly recognized as key determinants of vulnerability, clinical presentation, and treatment response in depression. Rather than arising from a single mechanism, these differences emerge from the interplay of multiple biological and non-biological factors. Converging evidence points to the hippocampus as a central region where these processes intersect, with adult neurogenesis and astrogliogenesis representing a potential mechanistic link between sex-specific biological factors and behavioral outcomes in depression. In this review, we integrate findings from human studies and preclinical models to examine how sex impacts depression while considering the multiple origins of sexual differentiation in the central nervous system. We discuss the importance of studying sex as a biological variable and acknowledge current limitations in the field. Finally, we highlight how cytogenic processes in the adult hippocampus are modulated in a sex-dependent manner, how their disruption may contribute to the pathophysiology of depression, and their potential role in precision psychiatry. Adult cytogenesis provides a promising target for developing therapeutic strategies aimed at promoting the integration of these cells in neural circuits, which may counterbalance the cellular impairments observed in stress-induced depression, representing a therapeutic avenue for this disorder.

## 1. Introduction

At the theoretical limits of medicine, in its fullest potential, the cure for every disease will likely rely on complete personalized treatments. While medicine has advanced toward this scenario in oncologic and rare genetic disorders, this degree of personalization remains limited in neuropsychiatric conditions [1,2]. This is due to the heterogeneous nature of mental illnesses, the reliance on subjective symptom-based diagnosis, and the biological barriers of the central nervous system [3]. These challenges hinder the development of reliable biomarkers and effective treatments, reinforcing the need for integrated and stepwise approaches combining imaging, electrophysiology, omics, genetics, and artificial intelligence toward personalized innovations [4,5]. Although full personalization remains elusive, accounting for biological sex represents a starting point for narrowing this gap. Beyond helping to elucidate the mechanisms that drive reported sex differences in cognition and behavior, considering sex may also improve our understanding of the factors that underlie differential brain responses in pathological conditions between males and females [6]. This is of particular interest in mental disorders such as major depressive disorder, characterized by sex-specific patterns in risk, prevalence, resilience mechanisms, coping strategies, and treatment response [7]. This condition imposes a substantial worldwide burden on individuals and healthcare systems, and the mechanism-based treatments available remain insufficient to effectively manage the complexity of the disease [4]. In this context, adult cytogenesis, the generation of new neurons and astrocytes in the adult brain, is emerging as a promising mechanism implicated not only in the pathophysiology of depression, but also in its treatment, with recent literature linking alterations in cellular plasticity to behavioral outcomes in depression [8].

In this review, we summarize the factors that may drive sex differentiation in the nervous system, as well as the multilevel sex-specific observations that may underlie epidemiologic patterns and differential treatment responses in depression. While sex differences have been documented across multiple domains, the influence of biological sex on astrogliogenesis remains virtually unexplored. We identify this as a critical gap, and we further propose and discuss how the inclusion of adult astrogliogenesis alongside neurogenesis may represent a potential personalized strategy aimed at ameliorating depression-related brain alterations. By integrating evidence across neuroendocrine, structural, and cellular levels, this review provides a multilevel framework for understanding the sex-dependent mechanisms underlying depression. Altogether, we aim to encourage further research into the role of biological sex in brain function, as its continued integration has the potential to improve diagnosis, drug development, and therapeutic strategies for brain disorders. Understanding these mechanisms may represent a critical step toward precision psychiatry. Throughout this review, we integrate evidence from human and preclinical studies, discuss areas of convergence and divergence, and highlight key translational gaps that may limit the interpretation and clinical relevance of mechanistic findings.

## 2. The Importance of Sex Differences in Science

It is becoming increasingly common to find references to both sexes in scientific articles, partly driven by the growing body of literature over recent years raising awareness of the importance of including biological sex as a variable when designing experiments and reporting data [9,10,11,12,13,14]. However, this has not always been the case, particularly in studies reporting the characterization of animal disease models, phenotypic behaviors, or mechanism-based drugs in which research tended toward the underrepresentation of females [11,15]. This paradigm started to change in 2001, when the National Academy of Medicine, formerly the Institute of Medicine of the National Academy of Sciences, stated that brain function exhibits important yet understudied sex differences in both physiologic and pathologic conditions, often to the detriment of the female brain [11]. In subsequent years, other agencies, together with the World Health Organization in 2008, endorsed the importance of studying the impact of sex [9]. However, despite these efforts, among animal studies in neuroscience published in 2009, only 20% included both sexes, while 42% failed to report the sex of their subjects [16]. In 2016, the National Institute of Health officially implemented policies requiring the inclusion of female subjects and the consideration of sex as a biological variable in all funded projects [10]. Indeed, this led to an immediate impact, with 52% of neuroscience studies in 2017 including both sexes, although 85% failed to disambiguate data by sex [16]. In 2019, a longitudinal analysis reported a 30% increase in studies including both sexes when compared to 2009, but only 19% reported optimal designs to assess possible sex differences [6]. Since then, these numbers have continued to rise, mainly driven by the efforts of major scientific journals, funding agencies, and editors that disseminate the integration of sex into research [13]. Researchers must also contribute to this effort by properly incorporating sex into their experimental design and data analysis, both as a covariate and dependent variable, while addressing some limitations. These include ensuring adequate sample sizes to account for variability; identifying the factors that make a trait different in males and females; suitable data analyses to discriminate the effects of other dependent variables of the study; and clear reporting of subjects used and sample sizes when journal publishing [6,11,15]. For detailed methodological guidance on how to study sex differences in neuroscience, see [11,17].

The major drawbacks of the underrepresentation of females in preclinical studies and clinical trials include disparities in the treatment and diagnosis of women; the perpetuation of male-biased knowledge; and the reduced reproducibility of findings using experimental protocols optimized in males [6]. Altogether, we are losing relevant information on (1) pathological nuances of several diseases that could help reduce adverse drug reactions in women, (2) molecules that may lead to vulnerability or resilience to disease, and (3) sex-specific fundamental mechanisms that could help the understanding of the human brain.

Indeed, while many similarities exist between men and women, strong evidence demonstrates robust sex differences in both normal and pathologic conditions, from brain development to aging [11]. One brain region particularly notable for sex-specific characteristics is the hippocampus, one of the most studied regions in neuroscience, and where differences have been reported in cognitive tasks and emotional responses relevant to learning and memory, as well as in neuroplastic mechanisms such as adult cytogenesis, a process by which new cells are generated in the adult brain [18]. In the case of sexually dimorphic diseases, where one sex is protected more than the other, women are more likely to develop mental health conditions such as depression, whereas men are more prone to neurodevelopmental disorders including autism spectrum disorder [11]. Moreover, even in diseases without pronounced sex differences in prevalence, the age of onset, time to diagnosis, disease progression, and treatment efficacy can differ between sexes [6].

Altogether, recognizing sex differences in brain function, disease vulnerability, and treatment response offers a critical opportunity to understand underlying mechanisms, identify protective factors and discover new therapeutic targets, which is essential to step psychiatry from symptom-based to mechanism-based treatments.

## 3. Origins of Sex Differentiation in the Nervous System

Sex differences in the human brain likely arise from the interplay of three main factors influencing the central nervous system from early development to adulthood: chromosomal sex, gonadal hormones, and sociocultural determinants.

### 3.1. Chromosomal Sex

Underlying many sex-specific brain characteristics is the fact that every neuron, astrocyte, and any other cell carries 22 pairs of autosomal chromosomes along with a male (XY) or female (XX) sex chromosome [11,19]. Interestingly, even in primary neuronal cultures, where system complexity is reduced and cells are not influenced by circulating hormones, we can still observe sex-related effects in dendritic morphogenesis and cellular complexity [20]. Although some genetic determinants may arise from sex-specific patterns of autosomal gene expression, most differences are thought to be linked to sex chromosomes [19,21]. The Y chromosome is relatively small and encodes few genes, whereas the X chromosome is large and gene-rich [11]. Early in embryogenesis, one of the two XX chromosomes is transcriptionally inactivated in all cells, leading to the silencing of most of its genes [11]. Although X-chromosome inactivation prevents most X-linked genes from being expressed, some may escape inactivation or be subjected to epigenetic modulation [22], leading to sex differences in gene expression in early brain development [23,24,25] and adulthood [21]. These transcriptomic differences can contribute to the sex-dependent organization of the central nervous system, which may later be further modulated by the action of adult gonadal hormones, ultimately influencing sex-specific vulnerability or protection to disease [11].

### 3.2. Gonadal Hormones

In humans, the XY genotype drives differentiation of the embryonic gonads into testes, whereas a XX genotype promotes ovarian differentiation [26]. Hormones produced by these organs subsequently guide sexual differentiation of the nervous system, and continue to influence multiple neural mechanisms in adulthood [26]. Gonadal hormones are steroid molecules derived from cholesterol and are broadly classified into androgens, typically associated with male characteristics, and estrogens and progesterone, usually associated with females [27]. The testes produce mostly the androgen testosterone, while ovaries produce mostly progesterone and an estrogen, 17-β estradiol [27]. Notably, progesterone serves as a precursor of testosterone, which in turn is the direct precursor of estradiol [27]. This interconnection between gonadal hormones is a good example of why their actions in the central nervous system are not straightforward. Remarkably, studies have shown that aromatase, the enzyme that converts testosterone to estrogen in the ovaries, is also present in multiple regions of the male brain. Notably, locally synthesized estrogen in the hippocampal dentate gyrus modulates synaptic plasticity and neurogenesis [28], a process enabled by measurable aromatase activity present in this region [28,29]. Indeed, although circulating testosterone exerts multiple direct effects, many of its neuronal and male-typical behavioral effects are thought to arise following its aromatization into estrogen [30].

In adulthood, this neuroendocrine system is mainly regulated by the hypothalamic–pituitary–gonadal (HPG) axis [31]. When activated, the hypothalamus releases gonadotropin-releasing hormone (GnRH), which stimulates the pituitary gland to produce luteinizing hormone (LH) and follicle-stimulating hormone (FSH) by the pituitary gland, ultimately inducing the production of gonadal hormones [31]. Given the hydrophobic nature of these molecules, gonadal hormones readily diffuse across cell membranes and bind to intracellular receptors, acting as transcription factors that bind to specific sites in the genome and modulate gene expression impacting both short- and long-term cell metabolism [27]. This modulation starts to occur during embryogenesis and postnatally, first organizing genitalia and brain regions, and later during adulthood by activating reversible physiological and behavioral responses [32].

Hormone levels fluctuate in cyclical patterns over time in the adulthood of both humans and rodents. Since these molecules have widespread effects on brain function, this variation may either enhance or mask sex-specific phenomena, which has hindered the study of sex differences in neuroscience for many years, often to the detriment of the female brain [11,33]. This was largely driven by the belief that including females introduced biological variability and reduced the statistical power of the analysis [33]. While the literature is increasingly agreeing that females are not more variable than males, as they do not display sign of variability in many physiological, morphological, or behavioral traits [34,35], controversy persists over the underlying reasons. Some studies show that hormonal oscillations during the estrous cycle do not influence depressive and anxious-like behaviors [33,36], whereas others show the opposite, with proestrus female rodents expressing less anxious-like phenotypes than females in the other estrous phases [37]. Nevertheless, the overall inclusion of females in studies is feasible, and whether these oscillations are relevant to the variables being studied should be addressed on a case-specific basis.

A reasonable approach is to include females, ignoring the possible effects of hormonal fluctuations, typically require larger sample sizes, and determining whether there is a sex difference in the variable being studied [11]. If the hormonal cycle has clear effects in the data, typically reflected by increased intragroup variability, one option is to assess hormonal status by hormonal sampling and later stratifying the data accordingly. Alternatively, this variable can be controlled by pharmacologically suppressing hormonal fluctuations or by performing gonadectomy. Additionally, studies show that standardizing tasks and the time of day at which experiments are performed, using age-matched animals, strictly avoiding olfactory cues from other animals in the experimental apparatus, and promoting long-term group housing of experimental animals help reduce interindividual variability [14]. These observations highlight that not only proper experimental design, but also effective colony management, can yield more reliable data [34,38].

A common misconception is that hormonal variability occurs only in the female estrous cycle and needs to be controlled only in females. However, human studies show that males also have diurnal fluctuations in testosterone and estrogen that can act as rapid neuromodulators in specific brain regions [39]. Consequently, controlling for hormonal influences in females only may be of limited value if similar male variability is ignored. Additionally, another layer of complexity is the fact that the nervous system is strongly influenced by gonadal hormones while controlling their synthesis [40], a process that can explain how sociocultural factors can shape sexual dimorphism at the neural level.

### 3.3. Sociocultural Determinants

Sociocultural factors contribute to sex differences in the brain by shaping brain development and influencing neuroplastic mechanisms in adulthood [41]. These non-biological factors, including early-life stressors, the historical roles of men and women in society, nuclear relationships, and daily life events, differentially impact males and females and can influence biological processes in a sex-specific manner [42].

Importantly, these sociocultural and biological influences are deeply intertwined: structural gender inequities, such as sex-based discrimination, wage gaps, and reduced access to resources, have been independently associated with higher rates of depression in women, even after controlling for individual-level factors [43].

In depression, studies have shown that sex-based social disparities are associated with higher rates of depression in females [43], while the lack of a partner is associated with higher rates in males [44].

Beyond relationship status, sex-specific stressor susceptibility plays an important role. While women show heightened reactivity to interpersonal and social rejection stressors, men are more vulnerable to occupational, financial, and legal stressors, reflecting how social roles translate into differential biological stress burden [43]. Furthermore, social support emerges as central psychosocial mediators of depression risk in females, whereas difficulties in emotion regulation are more prominent in males.

Additionally, early life stressors can induce molecular alterations that may drive sex-dependent susceptibility to stress later in life [45]. In this context, childhood sexual abuse, more prevalent in girls than boys, constitutes a potential risk factor, exerting neurobiological effects including endocrine stress response dysfunctions and epigenetic modifications, partly accounting for the higher incidence of depression in women [43]. Although the present review focuses on adulthood, it is important to acknowledge that sex-specific depression vulnerability might be in many cases developmentally programmed. The sex gap in depression may emerge during mid-puberty, driven by the interplay of activating sex hormones with intrapersonal susceptibility factors such as neuroticism and rumination as well as interpersonal stressors, converging biological and sociocultural factors that may shape differential risk trajectories into adulthood [43].

## 4. Sex Differences in Clinical Presentation of Depression

Major depressive disorder is one of the most common and debilitating forms of depressive disorder [46]. Currently, 330 million people worldwide suffer from depression [47], and the existing first-line therapies typically lead to around a 30–45% remission rate [48], highlighting this research field as a top priority.

These numbers have increased worldwide in recent years [49], and given that diagnostics and epidemiologic studies are not standardized across countries due to methodological limitations, these numbers may be underestimated [50]. Studies suggest that one contributing factor may be the fact that modern stressors are not of the same nature as those under which the human brain evolved [51]. Historically, humans developed adaptive responses to acute survival stressors such as avoiding predators and finding food, but today, stressors have shifted toward more chronic and psychological, such as persistent workplace pressure, economic and social demands, and daily life tempo, which may require different adaptive responses and ultimately impact individuals in a different manner [51].

Indeed, depression is a clear dimorphic pathology. Epidemiologically, women experience depression at twice the rate of men [52], an effect that emerges during puberty and persists until adulthood. The burden of disability is also higher in women with depression, who show earlier age of onset, lower quality of life, and greater comorbidity with anxiety and eating disorders [16,53]. Conversely, men present comorbidity with substance use disorders, and strikingly, self-harm and depression-related suicide rates are three times higher in men [7,54]. Coping mechanisms also differ between sexes, with women typically seeking psychiatric help more frequently, and reporting more internalizing symptoms [55,56].

Beyond epidemiology, sex differences also extend to treatment response, with evidence suggesting that women tend to respond more favorably to selective serotonin reuptake inhibitors, whereas men may show better outcomes with tricyclic antidepressants, despite some inconsistencies reflecting methodological limitations [57,58]. More recently, rapidly acting and long-lasting psychoactive compounds such as psilocybin have shown promising effects in alleviating depressive symptoms [59], with emerging preclinical studies indicating sex-dependent differences in its behavioral and neurobiological actions [60]. Psilocybin acutely increases head-twitch response frequency, with greater effects shown in females [61], and produces sex-specific behavioral responses to specific components of aversive stimuli [62].

Overall, depression is a heterogeneous disorder with pronounced sex differences in prevalence, comorbidity, and symptomatology. The biological and non-biological factors discussed earlier may explain at least some of this variability, and manifest as sex-specific, multilevel alterations, which are discussed in the following sections.

## 5. Multilevel Sex Differences in the Pathophysiology of Depression

Given the high variability of clinical manifestations, depression is characterized as a complex and heterogeneous disorder with substantial variability in pathophysiology, typically following exposure to stressful events [46]. This exposure to stressors triggers complex, multi-level responses that recruit neuroendocrine and behavioral systems to cope with the perturbation. While these acute adaptive responses are generally beneficial and essential for survival, their excessive or prolonged activation increases vulnerability to the development of depression [63].

Strikingly, individuals differ in how they perceive stressors. Most people experience numerous stressful events throughout their lifetime, yet only a small subset develops depression [64]. Moreover, comparable stressors can lead to different depressive outcomes across individuals [64]. Therefore, it is important to understand the neurobiological mechanisms that confer resilience and vulnerability to stress. Indeed, the link between neural dysfunctions, vulnerability, and behavioral responses is being progressively characterized, however, the factors that drive differences in these mechanisms between males and females are still to be identified.

### 5.1. Behavior

Sex differences in behavioral responses can be found in both human patients and in animal models of depression. In humans, male depression is typically presented with aggressivity, alcohol and substance use, escape, and risk-taking behaviors, while women show more affective symptoms, migraine attacks, hyperarousal episodes, appetite and sleep disturbances, guilt, depressed mood, and anxious episodes [16,53,65,66,67]. Interestingly, these male-typical manifestations are not explicitly described in the diagnostic criteria of the DSM-5, which may contribute to the higher reported prevalence of depression in women [67]. This may be due, in part, to a lower depression recognition in men, the fact that women are more likely to seek professional help for mental health problems, which has historically shaped the clinical presentation and diagnostic criteria used to define depression [43].

Although some depressive behaviors are uniquely human, animal models provide valuable insights into depressive-like symptoms and associated behaviors and reveal multiple sex-differential behavioral responses to stress-induced depression protocols [68]. Moreover, the same stress paradigm can impact different dimensions of behavior between males and females, with females being more resilient to cognitive alterations while more vulnerable to emotional changes [69]. Acquired learned-helplessness phenotypic behaviors are used to study coping strategies of animals when submitted to unavoidable stressful situations. A study found that an acute stressor can induce a depressive-like phenotype in the forced swim test in both male and female rats, while an unpredictable chronic mild stress protocol only induced depressive-like behaviors in males [70]. This may be explained either by a possible long-term protective role of estrogens in females [69] or be a consequence of consistent optimization of stress protocols in male subjects, which may result in reduced sensitivity to detect behavioral alterations in female populations [15]. Regarding cognition, females tend to show cognitive resilience to chronic stressors or even enhance their performance in cognitive tests such as the Morris water maze and the object placement test, while males often present learning and memory impairments [69]. In another study, female but not male mice also presented decreased self-care behavior in the sucrose splash test, upon subchronic variable stress, followed by an increase in serum corticosterone (CORT) levels [71]. Regarding anxiety, the results are more heterogeneous when referring to linear responses to stress, but different responses between males and females are also observed [72]. Other studies also report different coping strategies to stress between male and female rodents [73].

Despite the difficulty in tracing these behavioral differences to specific genetic or hormonal factors to date, since estrogens and progesterone affect emotions and cognition, it is believed that just as behavior reflects the integration of multiple cellular and molecular processes across distinct brain regions, the combinatorial effects of sex hormones are also thought to contribute to these differences [37].

### 5.2. Brain

Some of these behavioral disparities might be explained by structural and anatomical differences in specific regions of depressed brains [65]. High resolution imaging studies have been providing valuable data in recent years either by validating previous findings or by uncovering more subtle structural dimorphisms between males and females.

In adulthood, male brains are 10% to 15% larger than female brains [74], supported by different volumes of specific regions such as the amygdala, pallidum, and putamen, which are larger in males, and the prefrontal cortex, orbitofrontal cortex, and nucleus accumbens, which are larger in females [13,75]. Additionally, although human brains cannot be categorized into two distinct categories, male or female brain [76], studies suggest that male brains are structured to facilitate intrahemispheric communication while female brains favor interhemispheric communication [77]. Thus, there are likely to be many sexual dimorphisms in the human brain that become more pronounced in pathological conditions.

Structural alterations to the hippocampus constitute one of the most consistent findings regarding depression [65]. Physiologically, males and females tend to have similar sizes of the hippocampus [65,75,78], and volumetric reductions in this region are observed in patients with depression [7]. Sex might affect these volumetric changes, but whether this reduction is more prominent in males or females is not consistent across studies [7,65,79,80]. Another study also found sex-specific pattern abnormalities in prelimbic projections with prefrontal-limbic abnormalities being primarily found in women, while prefrontal-striatal abnormalities were primarily found in men [7,80]. Additionally, when submitted to acute psychosocial stressors, females with depression presented less deactivation of the amygdala, hippocampus, and nucleus accumbens when compared to healthy controls, an effect also not observed in males [65,81].

These findings may be of relevance to the differential clinical presentation of depression in women and men presented before. Prefrontal limbic dysfunctions may be correlated to more anxiety in women, while prefrontal striatal dysfunction to a higher prevalence of substance use in men, however, to date, no clear causality has been reported [7,82,83].

Some studies suggest that these sex specific differences in physiological and pathological conditions are largely due to the action of gonadal hormones during brain development, and their protective role in adulthood [32]. Indeed, estrogen has been highly associated with a protective role in the central nervous system, and this becomes more interesting when we look at studies reporting the effects of menopause. Menopause, a period of hormonal withdrawal in women, is associated with increased levels of anxiety, and sleep difficulties and is recognized as a vulnerable window to the development of depression [84,85]. Interestingly, a neuroimaging study using data from the UK Biobank reported that menopause is linked to reduced gray matter in the hippocampus, entorhinal cortex, and anterior cingulate cortex of women in post-menopause, an effect that was protected in women with ongoing hormone replacement therapy, particularly in the hippocampus [86].

### 5.3. HPA Axis

Coping with stress involves the action of the hypothalamic–pituitary–adrenal (HPA) axis, a central neuroendocrine system that physiologically responds to real or perceived stressors [87]. The HPA is activated in the hypothalamic paraventricular nucleus (PVN), which promotes the release of corticotropin-releasing hormone (CRH), vasopressin (AVP), and oxytocin (OT) which stimulates the release of adrenocorticotropin (ACTH) into the bloodstream by the anterior pituitary gland, and culminates in the secretion of glucocorticoids by the adrenal glands, cortisol in humans, and corticosterone in rodents [37,87]. These act upon virtually all tissues to facilitate a body-wide stress response. When acutely elevated by stressors, glucocorticoids induce beneficial physiological and behavioral changes necessary for the stress response [87,88]. However, prolonged elevations in glucocorticoids induced by chronic stress or disease states are detrimental and increase the risk for stress-related pathology [89]. In humans, findings on this topic are inconsistent, and the direction of the response does not appear to be linear, whereas rodent data provide more consistent patterns, though their translational relevance to human HPA dysregulation in depression requires careful interpretation [63]. In resting conditions, female rats have higher basal corticosterone than male rats [37,90]. Moreover, the response of females to stress is different from males. In response to several paradigms of acute stressors, corticosterone levels have been shown to be higher and to remain elevated for longer periods in females [87,90]. When returning to baseline activity, females also show a delayed response after acute stress, suggesting sex differences in the negative feedback regulation of the HPA axis [87]. Upon chronic stress paradigms, females also present higher elevations of basal corticosterone when compared to males [87,91]. Sex differences at each level of the HPA axis as well as in the involved limbic structures under stress may underlie these observations. Indeed, as stated before, some of the limbic structures that activate inhibitory inputs to the HPA axis, including the frontal cortex and the hippocampus, are differentially affected in males and females [90].

Moreover, the HPG axis activity can modulate and be modulated by the activity of the HPA axis, which may mediate some of the observed adult sex differences [40,92]. Studies show that in general, estradiol enhances the activity of the HPA axis [93]. Indeed, upon stress in female rats, the basal levels of the activity of the HPA axis follow those of estradiol throughout the phases of the estrous cycle, with higher activity in the estrous phase (high estradiol), and similar to males in the diestrus phase (low estradiol) [87]. Moreover, androgens generally inhibit the activity of the HPA axis. Thus, removal of most endogenous androgens by gonadectomy increases stress-induced ACTH and CORT secretion, whereas testosterone treatment has the opposite effects [87].

### 5.4. Cells

The alterations observed in depression are linked to impairments in cellular function. Depression is characterized by alterations in neuronal excitability and synaptic transmission in specific brain regions, often attributed to impaired neuronal [94] and astrocytic [95] function, as well as inflammatory markers typically associated with microglial dysfunction [51]. Moreover, these alterations also present sex-specific patterns [63].

The alterations in the neural circuitry observed in depression ultimately impact neuronal activity. In general, upon stress, the number of synapses is decreased in the hippocampus and the prefrontal cortex [94]. Morphological changes also occur upon stress with apical dendrites of CA3 pyramidal neurons atrophying [96], an effect observed in male rats, but not in females, that showed basal dendritic arbor atrophy [97]. In the medial prefrontal cortex, repeated restraint stress led to dendritic atrophy in males, but to a dendritic expansion in females [98]. This sex dependent response is further supported by the action of sex steroids in hippocampal synaptic transmission. Interestingly, a study found that acute 17β-estradiol treatment potentiates glutamatergic synaptic transmission in the hippocampus of males and females by increasing the strength of the synapses, but through different mechanisms in each sex [99].

Neuronal activity is highly influenced by neuron–astrocyte communication [100]. Indeed, glial pathology in depression is also well-documented by neuroanatomical studies on post-mortem brain tissues [95]. Moreover, neuroimaging studies report abnormal functional connectivity upon astrocyte dysfunction in preclinical models [101]. Studies also show that ablating glial cells in rodents is sufficient to induce depressive-like behaviors [102]. Chronic stress also induces sex-dependent astrocyte reactivity, with studies showing differential responses to the unpredictable chronic mild stress protocol between males and females, with females presenting overall higher astrocyte reactivity than males [103]. These alterations may be partly explained by astrocytic expression of glucocorticoid receptors [104].

Astrocyte reactivity is also influenced by microglial activation through the release of cytokines that modulate immune responses under both physiological and pathological conditions [103]. The role of inflammation in stress-related disorders is also well-established, with multiple molecular pathways of the immune system being activated upon stress and impacting neuronal circuits [51]. Microglia-mediated inflammation is also associated with sex differences. Studies show that upon stress, the number of microglial cells increases in the CA1 region of hippocampal females [105], and they also show a hypertrophic morphology [106].

Altogether, several studies show sex-specific dysfunction of multiple cells associated with depression in the hippocampus. Another relevant cellular mechanism occurring in this region also implicated in depression with sex-specific patterns is the generation of new cells in the adult brain, termed adult cytogenesis.

### 5.5. Adult Cytogenesis

The adult brain retains a remarkable capacity to generate new cells throughout life, a form of neural plasticity in which both new neurons (neurogenesis) and new astrocytes (astrogliogenesis) arise from neural stem cells within specialized cytogenic niches of the brain [107]. A critical limitation of the findings reviewed here concerns the methodological challenges of studying adult cytogenesis in living humans, which have generated substantial controversy in the field. Most mechanistic insights derive from rodent models, where neurogenesis can be quantified histologically using markers such as BrdU, Ki67, and DCX. In humans, however, the validity of DCX as a marker of newly born neurons in post-mortem tissue has been questioned, with studies reporting negligible neurogenesis in the adult human hippocampus [108], in direct contrast to findings demonstrating robust neurogenesis throughout adulthood [109]. These discrepancies likely reflect differences in tissue fixation protocols, post-mortem intervals, and antibody sensitivity, underscoring the need for methodological standardization and studies covering molecular and transcriptomic evidence for these cells. Indeed, despite these challenges, the presence of neural progenitors in the adult human brain is supported by modern transcriptomic studies [110] suggesting that cytogenesis, while difficult to quantify, remains a biologically relevant process in the human brain. Their generation has been shown to be crucial for cognitive and emotional domains of adult behavior, both in healthy and pathological contexts [109,111,112,113,114]. Indeed, studies indicate a relationship between stress and cytogenesis with sex- and experience-dependent outcomes. Beyond stress-induced suppression of neurogenesis [115], suppressing hippocampal cytogenesis sex-specifically disrupts the HPA axis activity [116], leading to connectivity impairments between the hippocampus and the prefrontal cortex, ultimately impacting emotional and cognitive behaviors [117]. Moreover, these processes were also shown to be regulated by steroid hormones [118].

#### 5.5.1. Adult Neurogenesis

Adult neurogenesis occurs in two main regions that generate distinct neuronal subtypes: the subgranular zone (SGZ) of the dentate gyrus (DG) in the hippocampus, originating glutamatergic neurons [110,119], and the subependymal zone (SEZ) of the lateral ventricles, which generates GABAergic interneurons for the olfactory bulb (OB) [119,120].

In humans, hippocampal adult-born neurons have been estimated to account for approximately 1400 new cells per day, corresponding to an annual turnover of around 1.75% of the neuronal population [120,121]. Upon generation, these cells may undergo maturation, migration, and functionally integration into the preexisting circuitry [122]. Importantly, this form of neuroplasticity is disrupted in numerous disease states [123]. Studies have shown that both patients and animal models of depression exhibit abnormalities in neurogenesis with impacts in behavioral outcomes such as cognition and emotional processing [124,125]. Post-mortem studies in depressed patients have revealed decreased cell proliferation in the hippocampal DG [126]. Similarly, under chronic social defect stress, neurogenic markers such as DCX and BrdU were decreased in the dentate gyrus of mice [124], and the survival and dendritic complexity of adult-born granule cells were also affected [127]. In line with these observations, unpredictable chronic mild stress lead to a decrease in the number of DCX, BrdU, and Ki67-positive cells in both the dorsal and ventral hippocampus [128,129,130].

When considering sex, the exposure to acute paradigms of stress is sufficient to reduce cell proliferation in male rats, but not in females [131]. Under chronic or repeated stress paradigms, another study reported opposite effects of hippocampal neurogenesis between males and females by decreasing in males while increasing in females [132]. Additionally, group housing was shown to be beneficial for females in coping with acute stressors by preventing the loss of proliferative cells, whereas an opposite effect was observed in males [133].

Moreover, antidepressant treatment and sex hormones were shown to modulate neurogenesis in the hippocampus with sex-specific patterns [113,134]. These sex-specific neurogenic responses provide a mechanistic complement to the clinical observations described before, where younger women showed greater response to the selective serotonin reuptake inhibitors (SSRIs) sertraline than older women and men [57]. SSRIs promote hippocampal neurogenesis partly through serotonin receptor-mediated stimulation of neural stem cell proliferation [135], a process that is further potentially amplified by estrogen signaling in females [57]. Conversely, the relative resistance of male hippocampal neurogenesis to antidepressant stimulation may contribute to the poorer SSRI response observed in men and the greater efficacy of tricyclic antidepressants, which have broader monoaminergic targets [57]. Finally, another factor linking sex to cytogenesis is epigenetic regulation. Sex-specific patterns of DNA methylation and histone modification have been identified in the hippocampus, where they directly regulate the expression of neurogenesis-related genes [136].

#### 5.5.2. Adult Astrogliogenesis

While the role of adult-born neurons has been extensively studied, the generation of adult-born astrocytes and their functional impact on networks remain comparatively unexplored. Nevertheless, astrogliogenesis plays multiple roles in brain function [112], and is increasingly recognized as an important process in pathological conditions [111].

In the adult brain, astrocytes can arise from at least two cell sources. The local division of mature differentiated astrocytes with self-renewal capabilities is visible in some regions of the cortex and the hippocampus and represents the primary source of newly generated astrocytes [137]. These locally dividing astrocytes can interact with the microenvironment and adapt their proliferation in response to stimuli [137], being particularly important upon tissue damage when the need for repopulation is high [138]. Secondly, adult astrocyte generation can also occur in the subgranular zone of the hippocampal dentate gyrus from neural stem cells together with adult-born neurons [112,139].

The proportion of adult-born astrocytes within the total pool of newly generated cells remains unclear, probably due to the lack of tools capable of labelling adult-born astrocytes in yields that resemble physiological conditions. Some studies estimated approximately 15% [95], while others reported substantially higher percentages, suggesting an approximately equal probability that a neural stem cell will generate either a neuron or an astrocyte [140]. Importantly, astrogliogenesis appears to be sensitive to both internal and external stimuli [112].

Although direct evidence for sex differences in astrogliogenesis remains limited, indirect evidence from the hormonal modulation of astrocytes provides possible mechanisms by which astrogliogenesis may also present sex-specific properties. Neural stem cells express estrogen receptors that respond to 17β-estradiol [141], comparable to mature astrocytes that express estrogen, androgen, and progesterone receptors, and respond to gonadal hormone fluctuations with changes in morphology, GFAP expression, glutamate uptake capacity, and neuroprotective function [142,143]. Glucocorticoids represent a second pathway with likely sex-differentiated effects. Studies show that the ablation of hippocampal cytogenesis produces sex-differentiated disruption of HPA axis activity, with male and female rats showing different basal corticosterone responses and distinct behavioral adaptations in response to the same cytogenic deficit [144].

Another study also found that transplanting glial-restricted precursor cells in a rat model of cytogenesis ablation reverts mood deficits and rescues hippocampal cytogenesis [145].

**Figure 1:**
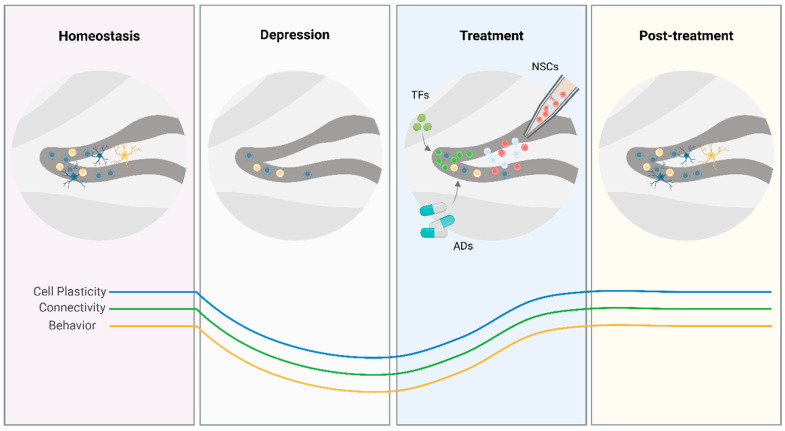
Dynamic changes in adult cytogenesis across homeostasis, depression, treatment, and recovery. Schematic representation of the temporal evolution of adult cytogenesis under physiological conditions (homeostasis), during depression, throughout treatment, and following treatment. Under homeostasis, balanced levels of neurogenesis and astrogliogenesis support normal brain function, including neural connectivity and behavior. In depression, cytogenic processes are disrupted, leading to reduced cell proliferation and impaired maturation and integration of newly generated cells, thereby altering brain plasticity, connectivity, and behavior. Treatment with antidepressants (ADs), transcription factors (TFs), or neural stem cell (NSC) transplantation is associated with a partial restoration of cytogenesis, promoting cell proliferation and functional integration into neural circuits. Post-treatment, cytogenic processes may stabilize toward baseline levels, contributing to the recovery of neural plasticity, brain connectivity, and behavior. This schematic summarizes current evidence in preclinical studies. Adapted from A Mateus-Pinheiro et al., 2013 [152], and created with BioRender^®^.

Altogether, these findings highlight adult cytogenesis as a critical mechanism of brain plasticity that is disrupted in stress-related disorders such as depression. Beyond their role in hippocampal circuitry and behavior, adult-born neurons and astrocytes respond to antidepressant treatment and are emerging as potential therapeutic targets in pathological conditions, paving the way for potential regenerative therapies [146]. Indeed, these findings suggest that preventing cytogenesis impairments, promoting the maturation and integration of adult-born cells, or restoring their numbers to physiologic levels either endogenously or by transplantation, may counterbalance the loss of mature neurons and astrocytes observed in stress-induced depression and other degenerative disorders, representing novel therapies (Figure 1) [147]. This is supported by findings applying such therapies in Huntington’s disease animal models. Huntington’s disease involves the selective loss of specific neural populations and progressive glial dysfunction, and studies show that human glial progenitor cells can outcompete and replace aged or diseased glia in the adult brain, effectively repopulating neural circuits and leading to measurable functional and molecular effects [148,149]. Future therapeutic strategies may move beyond pharmacological modulation toward more precise endogenous control of cytogenic fate. Transcription factors enhancing global cytogenesis or controlling a neurogenic-to-gliogenic switch, such as SOX9 and NFIA, which promote astrogliogenic commitment [150], and NGN2, which drives neuronal differentiation [151], represent compelling candidates, as their targeted manipulation could shift adult-born cell production toward the phenotype most depleted in a given pathological context.

However, realizing this therapeutic potential within a precision psychiatry framework remains in its early stages and requires reliable biomarkers capable of stratifying patients prior to initiating pro-neurogenic and/or pro-astrogliogenic interventions. In the future, non-invasive in vivo imaging tools, such as structural magnetic resonance imaging and positron emission tomography, might be utilized in the diagnosis of these conditions by evaluating hippocampal volume as an index of cytogenic activity in the dentate gyrus, while brain-derived extracellular vesicles represent an emerging peripheral alternative, potentially carrying molecular information reflective of active cytogenic processes.

Therefore, targeting adult cytogenesis of an individual may provide viable and highly personalized cell-based therapies for brain disorders in which cellular function is impaired.

## 6. Conclusions

**Figure 2:**
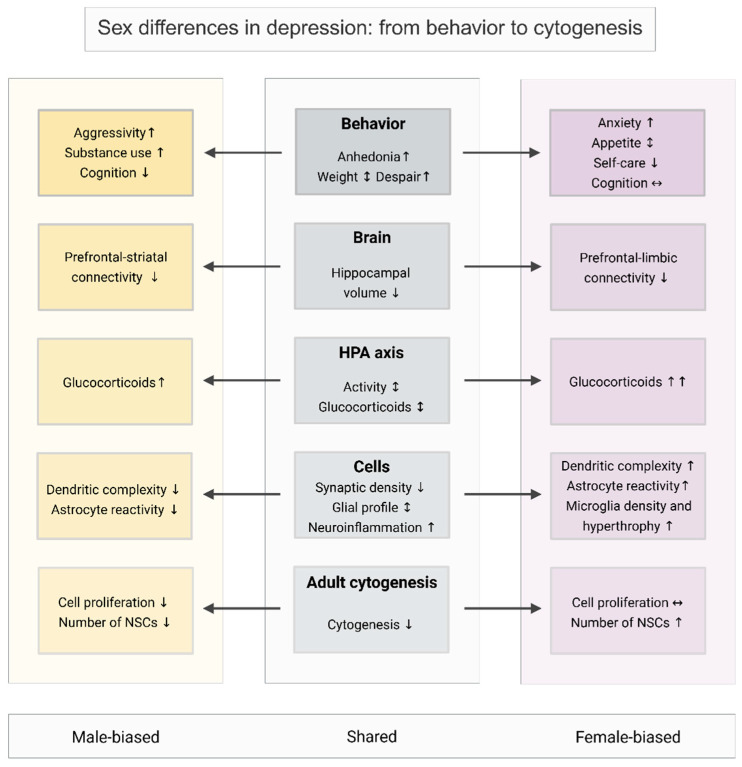
Summary of sex differences in depression. Conceptual overview of sex-biased and shared alterations across the behavioral, neural, neuroendocrine, cellular, and adult cytogenic domains. Male- and female-biased features are presented on opposite sides, with shared alterations centrally positioned. Arrows represent the direction and magnitude of change (↑ increase; ↑↑ stronger increase; ↓ decrease; ↔ no change; ↕ bi-directional changes). This schematic integrates evidence from human and preclinical studies and is intended as a conceptual summary of the evidence. Created with BioRender^®^.

The evidence reviewed here indicates that sex differences in depression emerge across multiple biological levels (Figure 2) including chromosomal and hormonal influences, HPA axis reactivity, brain structure, and cellular plasticity. These differences converge on the hippocampus and on adult cytogenesis, suggesting this region as a key mechanistic site through which biological sex influences vulnerability and resilience to depression.

Precision psychiatry represents a promising paradigm shift in mental healthcare, aiming to improve the currently available treatments by integrating individual clinical symptoms with biomarker signatures. In an ideal scenario, a single therapeutic strategy would be effective for all individuals. However, the biological heterogeneity of psychiatric disorders makes this unlikely to be true. This highlights the importance of incorporating sex as a biological variable into neuroscience research. Including both males and females in research is valuable, since studying them together helps optimize drugs for a broader population, while analyzing sexes separately can reveal sex-specific mechanisms for more targeted, mechanism-based therapies.

This is particularly relevant for depression where emerging approaches aimed at promoting brain repair, including pro-cytogenic and neural stem cell–based strategies, may offer new opportunities for regenerative and personalized treatments within the field of precision psychiatry [153]. Adult cytogenesis, including both neurogenesis and astrogliogenesis, has been identified as a key mechanism linking stress, cellular plasticity, and behavioral outcomes in depression. Nevertheless, several limitations remain. Most mechanistic insights into sex differences in adult cytogenesis derive from rodent models, and their direct translation to human biology requires further validation. In addition, causal relationships between cytogenic processes and depressive phenotypes are not fully established, and the contribution of astrogliogenesis remains comparatively underexplored. Part of the problem arises from the lack of tools to specifically target and modulate adult-born cellular populations and their progeny, with most studies relying on viral approaches and combinations of cell proliferation markers. The development of multicolor animal models and specific tools to manipulate or ablate adult-born neurons and astrocytes independently will enable more precise lineage tracing and clonal analysis, and ultimately help to resolve the distinct functional contributions of each population. Overall, the concept of adult cytogenesis as a potential target is also still evolving, with a need for standardized markers and reliable in vivo proxies.

Future research should focus on defining the molecular mechanisms linking sex hormones to cytogenic processes, developing translational biomarkers, and testing whether pro-cytogenic interventions can be tailored to sex-specific biological profiles. This will require the systematic inclusion of sex as a biological variable in both preclinical and clinical studies. Altogether, elucidating sex differences in brain function and disease mechanisms and whether they can be modulated in a beneficial way for human health, is a critical step toward advancing our understanding of depression and developing more targeted and effective therapies.

## Data Availability

No new data were created or analyzed in this study.
